# 4-Methylenesterols from a Sponge *Theonella swinhoei*

**DOI:** 10.3390/md10071536

**Published:** 2012-07-19

**Authors:** Jheng-Kun Guo, Ching-Ying Chiang, Mei-Chin Lu, Wen-Been Chang, Jui-Hsin Su

**Affiliations:** 1 National Museum of Marine Biology & Aquarium, Pingtung 944, Taiwan; Email: idun031177@hotmail.com (J.-K.G.); jinx6609@nmmba.gov.tw (M.-C.L.); 2 Graduate Institute of Marine Biodiversity and Evolutionary Biology, National Dong Hwa University, Pingtung 944, Taiwan; 3 Center of General Studies, National Kaohsiung Marine University, Kaohsiung 811, Taiwan; Email: cyjang@webmail.nkmu.edu.tw; 4 Graduate Institute of Marine Biotechnology, National Dong Hwa University, Pingtung 944, Taiwan; 5 Asia-Pacific Ocean Research Center, National Sun Yat-sen University, Kaohsiung 804, Taiwan

**Keywords:** 4-methylenesterols, sponge, *Theonella*

## Abstract

Three new 4-methylenesterols, theonellasterol K (**1**), acetyltheonellasterol (**2**) and acetyldehydroconicasterol (**3**), along with two known sterols, theonellasterol (**4**) and theonellasterone (**5**), were isolated from the sponge *Theonella swinhoei*. The structures of these compounds were elucidated on the basis of their spectroscopic data and comparison of the NMR data with those of known analogues. Compound **1** exhibited significant cytotoxic activity against HCT-116, K562 and Molt 4 cancer cell lines.

## 1. Introduction

**Figure 1:**
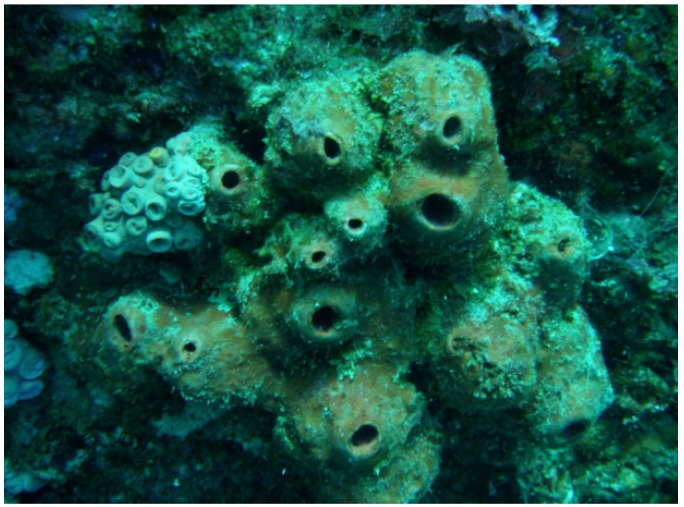
Sponge *Theonella swinhoei.*

**Chart 1:**
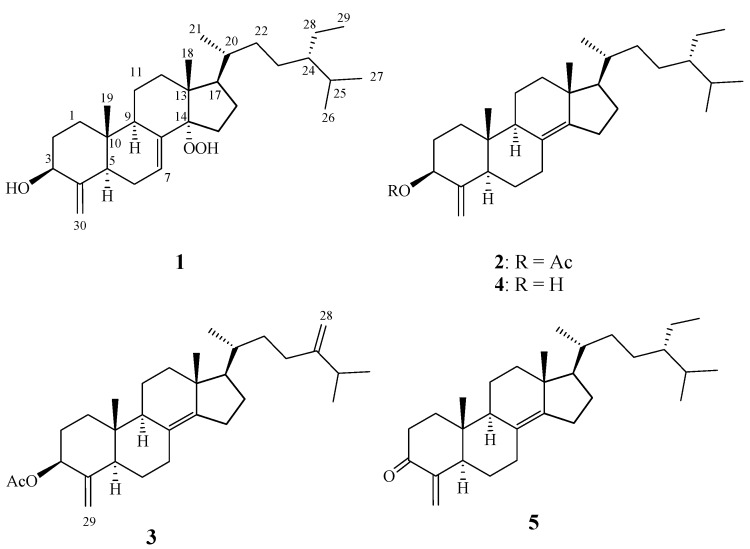
Structures of metabolites **1–5**.

In recent years, marine sponges have emerged as one of the most prolific sources for discovery of novel secondary metabolites [1,2,3]. Some of these have been found to possess several kinds of biological activities, such as cytotoxic [4,5], antimicrobial [6], and antiviral properties [7]. 4-Methylenesterols were first reported in 1981 as being obtained from two marine sponges, *Theonella conica* and *Theonella swinhoei*, from the Red Sea [8]. Marine sponges of the genus *Theonella* are now known to be a rich source of novel 4-methylene sterols [8,9,10,11,12,13,14,15,16,17,18,19,20,21]. Some of these sterols display a variety of biological activities, and pharmacological evaluation has shown them to be modulators of two well-known nuclear receptors, FXR and PXR [16,17,18,19,20,21], and cytotoxic activity [9]. Furthermore, a recently published report indicates that theonellasterol (**4**) is a highly selective FXR antagonist that protects against cholestatic liver injury [21]. Research into the pharmacological properties of this class of natural products is of particular interest. Our investigation of the chemical constituents of the sponge *Theonella swinhoei* (Figure 1) yielded three new 4-methylene sterols, theonellasterol K (**1**), acetyltheonellasterol (**2**) and acetyldehydroconicasterol (**3**), along with two known sterols, theonellasterol (**4**) [8] and theonellasterone (**5**) [10] (Chart 1). The structures of **1–5** were established by detailed spectroscopic analysis, including extensive examination of 2D NMR (^1^H-^1^H COSY, HMQC and HMBC) correlations. The cytotoxicity of metabolites **1–5** against human colorectal carcinoma (DLD-1), human hormone-dependent breast cancer (T-47D), human colon adenocarcinoma (HCT-116), human breast adenocarcinoma (MCF-7 and MDA-MB-231), human chronic myelogenous leukemia (K562) and human T lymphoblast, acute lymphoblastic leukemia (Molt 4) was studied in order to discover bioactive compounds.

## 2. Results and Discussion

The EtOAc extract of the freeze-dried specimen was fractionated by silica gel column chromatography and the eluted fractions were further separated utilizing normal phase HPLC to yield metabolites **1–5**. The new compounds were given the trivial names theonellasterol K (**1**), acetyltheonellasterol (**2**) and acetyldehydroconicasterol (**3**). The known compounds were identified as theonellasterol (**4**) and theonellasterone (**5**). 

**Table 1:** ^1^H and ^13^C NMR data for **1–3**.

	1		2		3	
	δ_H_ ( *J* in Hz) ^a^	δ_C_ (mult.) ^b^	δ_H_ ( *J* in Hz) ^a^	δ_C_ (mult.) ^b^	δ_H_ ( *J* in Hz) ^a^	δ_C_ (mult.) ^b^
1	1.39 m; 1.82 m	37.2 (CH_2_)	1.38 m; 1.80 m	36.5 (CH_2_)	1.38 m; 1.79 m	36.5 (CH_2_)
2	1.42 m; 1.98 m	32.7 (CH_2_)	1.50 m; 1.94 m	29.6 (CH_2_)	1.52 m; 1.94 m	29.6 (CH_2_)
3	4.01 dd (11.0, 5.0)	73.2 (CH)	5.15 dd (12.0, 5.0)	74.8 (CH)	5.15 dd (12.0, 5.0)	74.8 (CH)
4		151.9 (C)		148.0 (C)		148.0 (C)
5	1.97 m	45.1 (CH)	1.89 d (13.0)	49.5 (CH)	1.90 m	49.5 (CH)
6	2.07 m; 2.14 m	25.1 (CH_2_)	1.38 m; 1.84 m	27.0 (CH_2_)	1.40 m; 1.84 m	27.0 (CH_2_)
7	5.68 d (5.5)	125.0 (CH)	2.25 m	25.8 (CH_2_)	2.25 m	25.8 (CH_2_)
8		135.5 (C)		125.5 (C)		125.6 (C)
9	2.17 m	45.4 (CH)	1.80 m	49.1 (CH)	1.80 m	49.1 (CH)
10		37.2 (C)		39.8 (C)		39.8 (C)
11	1.47 m; 1.64 m	20.8 (CH_2_)	1.48 m; 1.64 m	20.4 (CH_2_)	1.48 m; 1.64 m	20.4 (CH_2_)
12	1.62 m; 1.82 m	30.5 (CH_2_)	1.14 m; 1.96 m	37.3 (CH_2_)	1.15 m; 1.96 m	37.3 (CH_2_)
13		47.3 (C)		42.7 (C)		42.8 (C)
14		98.3 (C)		143.1 (C)		143.0 (C)
15	1.34 m;	26.9 (CH_2_)	1.75 m;	29.2 (CH_2_)	1.77 m;	29.2 (CH_2_)
	2.06 m		2.47 dd (13.5, 1.0)		2.47 d (14.0)	
16	1.62 m; 2.08 m	24.8 (CH_2_)	1.36 m; 1.60 m	24.5 (CH_2_)	1.36 m; 1.59 m	24.5 (CH_2_)
17	1.85 m	50.9 (CH)	1.16 m	56.7 (CH)	1.17 m	56.7 (CH)
18	0.74 s	17.0 (CH_3_)	0.84 s	18.2 (CH_3_)	0.85 s	18.2 (CH_3_)
19	0.71 s	13.6 (CH_3_)	0.62 s	13.1 (CH_3_)	0.62 s	13.1 (CH_3_)
20	1.37 m	36.3 (CH)	1.46 m	34.9 (CH)	1.50 m	34.4 (CH)
21	0.89 d (7.0)	19.0 (CH_3_)	0.95 d (6.5)	19.2 (CH_3_)	0.96 d (6.5)	19.1 (CH_3_)
22	1.00 m; 1.40 m	34.0 (CH_2_)	1.06 m; 1.41 m	33.7 (CH_2_)	1.24 m; 1.60 m	34.4 (CH_2_)
23	1.04 m; 1.34 m	26.7 (CH_2_)	1.04 m; 1.32 m	26.2 (CH_2_)	1.90 m; 2.08 m	30.8 (CH_2_)
24	0.94 m	46.0 (CH)	0.92 m	46.1 (CH)		156.9 (C)
25	1.68 m	29.0 (CH)	1.68 m	28.9 (CH)	2.24 m	33.8 (CH)
26	0.81 d (7.0)	18.9 (CH_3_)	0.82 d (8.5)	19.0 (CH_3_)	1.03 d (7.0)	21.9 (CH_3_)
27	0.83 d (7.0)	19.6 (CH_3_)	0.83 d (7.0)	19.5 (CH_3_)	1.04 d (7.5)	22.0 (CH_3_)
28	1.14 m; 1.32 m	23.0 (CH_2_)	1.16 m; 1.32 m	23.0 (CH_2_)	4.67 s; 4.73 s	105.9 (CH_2_)
29	0.86 t (7.5)	12.3 (CH_3_)	0.86 t (7.5)	12.3 (CH_3_)	4.60 s; 4.89 s	103.7 (CH_2_)
30	4.74 s; 5.18 s	103.6 (CH_2_)	4.60 s; 4.89 s	103.7 (CH_2_)		
3-OAc			2.13 s	21.2 (CH_3_)	2.13 s	21.2 (CH_3_)
				170.2 (C)		170.2 (C)
14-OOH	6.79 br s					

^a^ 500 MHz in CDCl_3_; ^b^ 125 MHz in CDCl_3_; ^c^ *J* values (Hz) are given in parentheses; ^d^ Numbers of attached protons were deduced by DEPT experiments.

**Figure 2:**
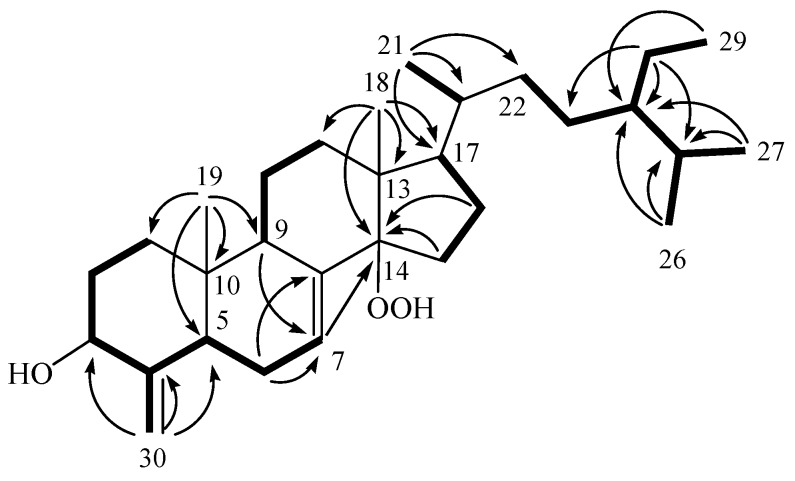
Selected ^1^H-^1^H COSY (▬) and HMBC (→) correlations of **1**.

Theonellasterol K (**1**) was obtained as a white amorphous solid. The HRESIMS spectrum of **1** exhibited a pseudomolecular ion peak at *m*/*z* 481.3659 [M + Na]^+^, which established a molecular formula of C_30_H_50_O_3_, implying six degrees of unsaturation. IR absorptions were observed at 3362 and 3251 cm^−1^, suggesting the presence of hydroxy groups in **1**. The structure of this compound was deduced from its ^13^C NMR and DEPT spectroscopic data (Table 1), which showed that the compound has 30 carbons, including six methyls, ten sp^3^ methylenes, one sp^2^ methylene, seven sp^3^ methines (including one oxymethine), one sp^2^ methine, three sp^3^ quaternary carbons and two sp^2^ quaternary carbons. From the ^1^H NMR spectrum of **1**, resonances of one olefinic methine proton (δ 5.68, d, *J* = 5.5 Hz), two olefinic methylene protons (δ 5.18 and 4.74, each s) and one oxygenated methine (δ 4.01, dd, *J* = 11.0, 5.0 Hz) were observed. Moreover, the ^1^H NMR spectrum revealed the presence of one hydroperoxy proton resonating as a broad singlet at δ_H_ 6.79. The planar structure and all of the ^1^H and ^13^C chemical shifts of **1** were elucidated by 2D NMR spectroscopic analysis, in particular ^1^H-^1^H COSY and HMBC experiments (Figure 2). From the ^1^H-^1^H COSY correlations, it was found that one ring-juncture methine proton H-5 (δ 1.97) and one oxymethine proton H-3 (δ 4.01) exhibited allylic correlations with the exomethylene protons at C-30 (δ_H_ 4.74 and 5.18). In addition, ^1^H-^1^H COSY spectral analysis established five partial structures of consecutive proton spin systems (Figure 2). Further analysis of the HMBC correlations was employed successfully to establish the gross structure of **1** (Figure 2). Thus, **1** was found to possess two double bonds at C-7/C-8 and C-4/C-30, one hydroxy group at C-3 and one hydroperoxy group at C-14.

**Figure 3:**
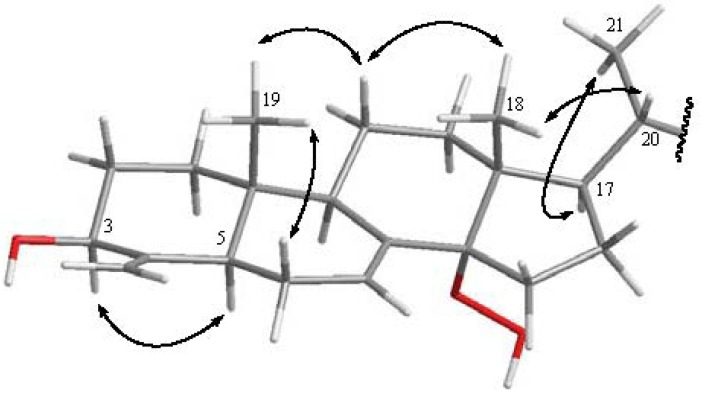
Computer-generated model of **1** using MM2 force field calculations and selected NOE correlations from C-1–C-21 of **1**.

The relative configuration of **1**, elucidated mainly from the NOESY spectrum, was compatible with that of **1** ascertained using molecular mechanics calculations (MM2), which suggested the most stable conformations, as shown in Figure 3. In the NOESY spectrum of **1**, the NOE correlations between one of the methylene protons at C-11 (δ_H_ 1.47) and both methyls (H_3_-18 and H_3_-19); H_3_-18 and H-20 as well as between H_3_-18, H_3_-19 and H-20 indicated that these protons adapt a *β*-orientation. H-5 was found to interact with H-3, but not with H_3_-19, revealing the *β* orientation of the hydroxy group at C-3. Moreover, the *α*-orientation of 14-OOH was further confirmed by the lower field chemical shift of H-17 (δ_H_ 1.85, m). Furthermore, the chemical shifts of the side chain from C-20 to C-29 in **1** were nearly identical to those of **4** and **5**. Thus, the structure of steroid **1** was established. After determining the structure of **1**, we discovered that its molecular framework has been obtained as a known 4-methylenesterol conicasterol H, which was isolated previously from sponge *Theonella swinhoei* [18].

Acetyltheonellasterol (**2**) was isolated as a white powder with the molecular formula C_32_H_52_O_2_, which possesses seven degrees of unsaturation, as indicated by HRESIMS (*m*/*z* 491.3862, [M + Na]^+^) and NMR spectroscopic data (Table 1). By comparison of the NMR data of **2** with those of **4**, it was found that the ^1^H and ^13^C NMR data of **2** were very similar to those of **4**, with the difference that **2** contains one more acetyl group relative to **4**. The chemical shift of H-3 in **4** (δ_H_ 4.01) was shifted downfield (δ_H_ 5.15) in **2**, suggesting that **2** is the 3-acetyl derivative of **4**. We observed further that acetylation of **4** gave a product which was found to be identical to **2** by comparison of the physical and spectroscopic data. Thus, compound **2** was established as the 3-acetyl derivative of **4**.

The new metabolite acetyldehydroconicasterol (**3**) was obtained as a white powder and possessed the molecular formula C_31_H_48_O_2_, as established from the HRESIMS and NMR data, implying eight degrees of unsaturation. Both the ^1^H and ^13^C NMR signals of **3** were found to be very closely related to those of compound **2**, suggesting a very similar steroidal skeleton. By comparison of the NMR data of **3** with those of **2** (Table 1), it was found that an ethyl protons signal [δ_H_ 0.86 t, *J* = 7.5 Hz (H_3_-29); 1.16 m and 1.32 m (H_2_-28)] in **2** was replaced by two exomethylene proton signals (δ_H_ 4.73 and 4.67, each s) in **3**. The only difference observed was that the 24-ethyl side chain of **2** was replaced by two exomethylene proton signals in **3**. This was further confirmed by the HMBC correlations from H_2_-28 to C-23, C-24, and C-25. On the basis of the above analysis, the structure of **3** was established. 

Finally, we used a 3-(4,5-dimethylthiazol-2-yl)-2,5-diphenyl tetrazolium bromide (MTT) assay to examine the cytotoxic activities of compounds **1–5** against DLD-1, T47D, HCT-116, MCF-7, MDA-MB-231, K562 and Molt 4 cancer cells. Cells were treated with different concentrations of **1–5** for 72 h. The results showed that compound **1**, the most potent of compounds **1–5**, exhibited cytotoxicity against DLD-1, T47D, HCT-116, MDA-MB-231, K562 and Molt 4, with IC_50_s of 12.9, 12.0, 6.3, 11.5, 11.4, 4.3, and 6.3 μg/mL, respectively. Furthermore, compound **2** exhibited weak cytotoxic activity against K562 and Molt 4 cancer cell lines (the IC_50_ values were 13.7 and 17.8 μg/mL for K562 and Molt 4, respectively). The other tested compounds were not cytotoxic (IC_50_ > 20 μg/mL) towards the above seven cancer cell lines (Table 2).

**Table 2:** Cytotoxicity (IC_50_ μg/mL) of compounds **1–5** ^a^.

	Cell Lines						
	DLD-1	T-47D	HCT-116	MCF-7	MDA-MB-231	K562	Molt 4
**1**	12.9	12.0	6.3	11.5	11.4	4.3	6.3
**2**	– ^c^	– ^c^	– ^c^	– ^c^	– ^c^	13.7	17.8
**Doxorubicin ^b^**	0.42	0.28	0.89	2.2	1.3	0.14	0.009

^a^ Compounds **3–5** was inactive against all seven cell lines (IC_50_ > 20 μg/mL); *^b^* Clinical anticancer drug used as a positive control; ^c^ NA, not active at 20 μg/mL.

## 3. Experimental Section

### 3.1. General Experimental Procedures

Optical rotation values were measured with a Jasco P-1010 digital polarimeter. IR spectra were recorded on a Varian Digilab FTS 1000 Fourier transform infrared spectrophotometer. The NMR spectra were recorded on a Varian Mercury Plus 400 FT-NMR (or Varian Unity INOVA 500 FT-NMR) instrument at 400 MHz (or 500 MHz) for ^1^H-NMR and 100 MHz (or 125 MHz) for ^13^C-NMR, respectively, in CDCl_3_. ESIMS were obtained with a Bruker APEX II mass spectrometer. Gravity column chromatography was performed on silica gel (230–400 mesh, Merck). TLC was carried out on precoated Kieselgel 60 F254 (0.2 mm, Merck) and spots were visualized by spraying with 10% H_2_SO_4_ solution followed by heating. High-performance liquid chromatography (HPLC) was performed using a system comprised of a Hitachi L-7100 pump and a Rheodyne 7725 injection port. A preparative normal phase column (250 × 21.2 mm, 5 μm) was used for HPLC.

### 3.2. Animal Material

The specimen of *Theonella swinhoei* was collected by scuba divers at a depth of 15–20 m from coral reefs off the coast of Pingtung, Taiwan. A voucher specimen was deposited in the National Museum of Marine Biology and Aquarium, Taiwan (specimen no. 2011SP-2). Taxonomic identification was performed by Prof. Wen-Been Chang of the National Museum of Marine Biology & Aquarium, Pingtung, Taiwan.

### 3.3. Extraction and Separation

The sponge *Theonella swinhoei* (2.2 kg fresh wt) stored frozen and then freeze dried. The freeze-dried material (590 g) was minced and extracted exhaustively with EtOAc (5 × 2 L). The EtOAc extract was evaporated to yield a residue (11.9 g), which was subjected to open column chromatography on silica gel eluting with *n*-hexane (H)-EtOAc (E) gradient and EtOAc (E)-acetone (A) gradient, to give 12 fractions: Fr-1 (eluted by H-E 100:1), Fr-2 (eluted by H-E 50:1), Fr-3 (eluted by H-E 30:1), Fr-4 (eluted by H-E 20:1), Fr-5 (eluted by H-E 10:1), Fr-6 (eluted by H-E 8:1), Fr-7 (eluted by H-E 5:1), Fr-8 (eluted by H-E 3:1), Fr-9 (eluted by H-E 1:1), Fr-10 (eluted by EtOAc), Fr-11 (eluted by E-A 1:1) and Fr-12 (eluted by acetone). Fraction 2 (250 mg), was subjected to normal phase HPLC (Hibar 250 × 21.2 mm, Supelco, silica gel 60, 5 μm), using *n*-hexane–EtOAc (25:1) as eluent, to afford four subfractions (A1–A4). Subfraction A1 (50 mg) was separated by normal phase HPLC using *n*-hexane–EtOAc (25:1) to afford **2** (25.5 mg, 0.21% dry wt of extract) and **3** (1.8 mg, 0.015% dry wt of extract). Subfraction A3 (120 mg) was also purified by normal phase HPLC using *n*-hexane–EtOAc (15:1) to afford **5** (55 mg, 0.46% dry wt of extract). Fraction 5 (2.5 g), was further separated by silica gel open column chromatography with gradient elution (*n*-hexane–EtOAc, 10:1 to 5:1) to afford **4** (1.2 g, 10.1% dry wt of extract). Fraction 7 (250 mg), was further separated by normal phase HPLC (*n*-hexane–EtOAc, 5:1) to yield six subfractions (B1–B6). Subfraction B3 (30 mg) was separated by normal phase HPLC using *n*-hexane–acetone (6:1) to afford **1** (12.5 mg, 0.11% dry wt of extract). 

Theonellasterol K (**1**): white powder; [α]^24^_D_ = +82 (*c* 0.2, CHCl_3_); IR (neat) ν_max_ 3362, 3251, 2956, 2870, 1644, 1451 and 1376 cm^−1^; ^1^H and ^13^C NMR data, see Table 1; ESIMS *m*/*z* 481 [50, (M + Na)^+^]; HRESIMS *m*/*z* 481.3659 (calcd. for C_30_H_50_O_3_Na, 481.3657).

Acetyltheonellasterol (**2**): white powder; [α]^24^_D_ = −3 (*c* 1.0, CHCl_3_); IR (neat) ν_max_ 2957, 1744, 1644, and 1373 cm^−1^; ^1^H and ^13^C NMR data, see Table 1; ESIMS *m*/*z* 491 [80, (M + Na)^+^]; HRESIMS *m*/*z* 491.3862 (calcd. for C_32_H_52_O_2_Na, 491.3865).

Acetyldehydroconicasterol (**3**): white powder; [α]^24^_D_ = −5 (*c* 0.1, CHCl_3_); IR (neat) ν_max_ 2927, 1742, and 1376 cm^−1^; ^1^H and ^13^C NMR data, see Table 1; ESIMS *m*/*z* 475 [100, (M + Na)^+^]; HRESIMS *m*/*z* 475.3555 (calcd. for C_31_H_48_O_2_Na, 475.3552).

Acetylation of **4**: A solution of **4** (10.0 mg) in pyridine (0.2 mL) was mixed with Ac_2_O (0.2 mL), and the mixture was stirred at rt for 24 h. After evaporation of excess reagent, the residue was subjected to column chromatography over Si gel using *n*-hexane–EtOAc (7:1) to yield **2** (10.5 mg, 95%). The specific rotation [[α]^24^_D_ = −3 (*c* 0.5, CHCl_3_)] was in full agreement with that of the natural product **2**.

### 3.4. Cytotoxicity Testing

Cell lines were purchased from the American Type Culture Collection (ATCC). Cytotoxicity assays of compounds **1–5** were performed using the MTT [3-(4,5-dimethylthiazol-2-yl)-2,5-diphenyltetrazolium bromide] colorimetric method [22,23]. 

### 3.5. Molecular Mechanics Calculations

Implementation of the MM2 force filed in Chem3D Pro software [24] was used to calculate the molecular models.

## 4. Conclusions

A series of new 4-methylenesterols were isolated from the sponge *Theonella swinhoei* [8,9,10,11,12,13,14,15,16,17,18,19,20,21]. Our continued investigation of the chemical constituents of sponge *T. swinhoei* has again led to the isolation of three new 4-methylenesterols (**1–3**) and two known 4-methylenesterols (**4** and **5**). Compound **1** exhibited significant cytotoxicity against HCT-116, K562 and Molt 4 cells, and moderate to weak cytotoxicity against DLD-1, MCF-7, and MDA-MB-231 cells. In addition, compound **2** exhibited weak cytotoxicity toward K562 and Molt 4 cell lines. However, the new compound **3** and the other known compounds had no significant activity. According to our research, metabolite **1** exhibited significant cancer cells inhibitory activity. This result suggests that **1** should be subjected to further biomedical investigation.

## Supplementary Files

Supplementary File 1: PDF-Document (PDF, 3908 KB)
