# Supplementary material for: 4-Methylenesterols from a Sponge Theonella swinhoei
Source: Mar Drugs. 2012 Jul 19;10(7):1536–44. doi: 10.3390/md10071536 (PMC3407929; doi:10.3390/md10071536)
Supplement: Supplementary File 1: — PDF-Document (PDF, 3908 KB) [file marinedrugs-10-01536-s001.pdf]

Supporting Information

4-Methylenesterols from a Sponge *Theonella swinhoei*

Table of Contents

S1. Table of Contents

S2. ^{1}H NMR spectrum of **1** in $CDCl_3$ at 500 MHz.

S3. ^{13}C NMR spectrum of **1** in $CDCl_3$ at 125 MHz.

S4. ^{1}H NMR spectrum of **2** in $CDCl_3$ at 500 MHz.

S5. ^{13}C NMR spectrum of **2** in $CDCl_3$ at 125 MHz.

S6. ^{1}H NMR spectrum of **3** in $CDCl_3$ at 500 MHz.

S7. ^{13}C NMR spectrum of **3** in $CDCl_3$ at 125 MHz.

S8. ^{1}H NMR spectrum of **4** in $CDCl_3$ at 400 MHz.

S9. ^{13}C NMR spectrum of **4** in $CDCl_3$ at 100 MHz.

S10. ^{1}H NMR spectrum of **5** in $CDCl_3$ at 400 MHz.

S11. ^{13}C NMR spectrum of **5** in $CDCl_3$ at 100 MHz.

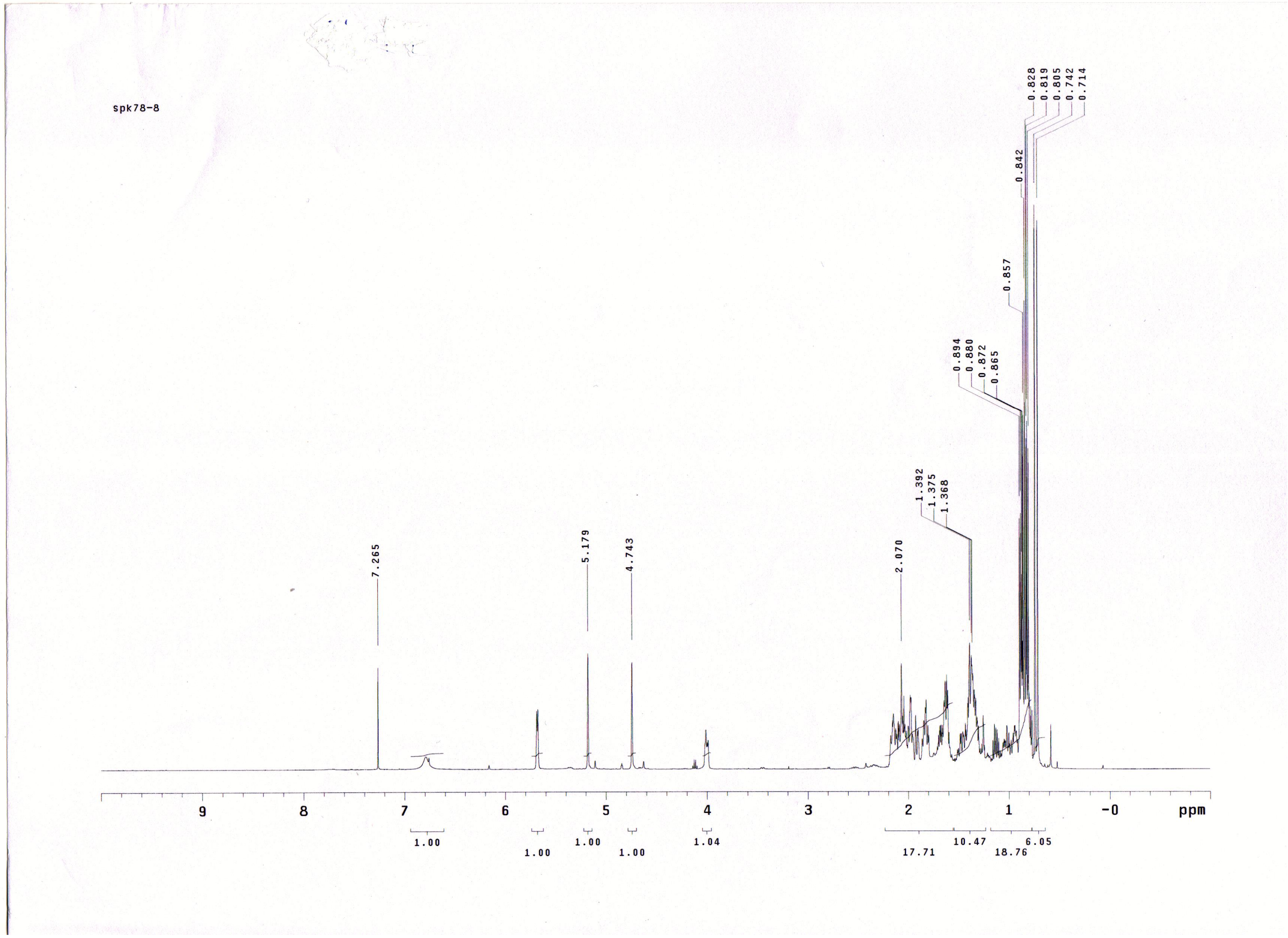

S2. ^{1}H NMR spectrum of **1** in $CDCl_3$ at 500 MHz.

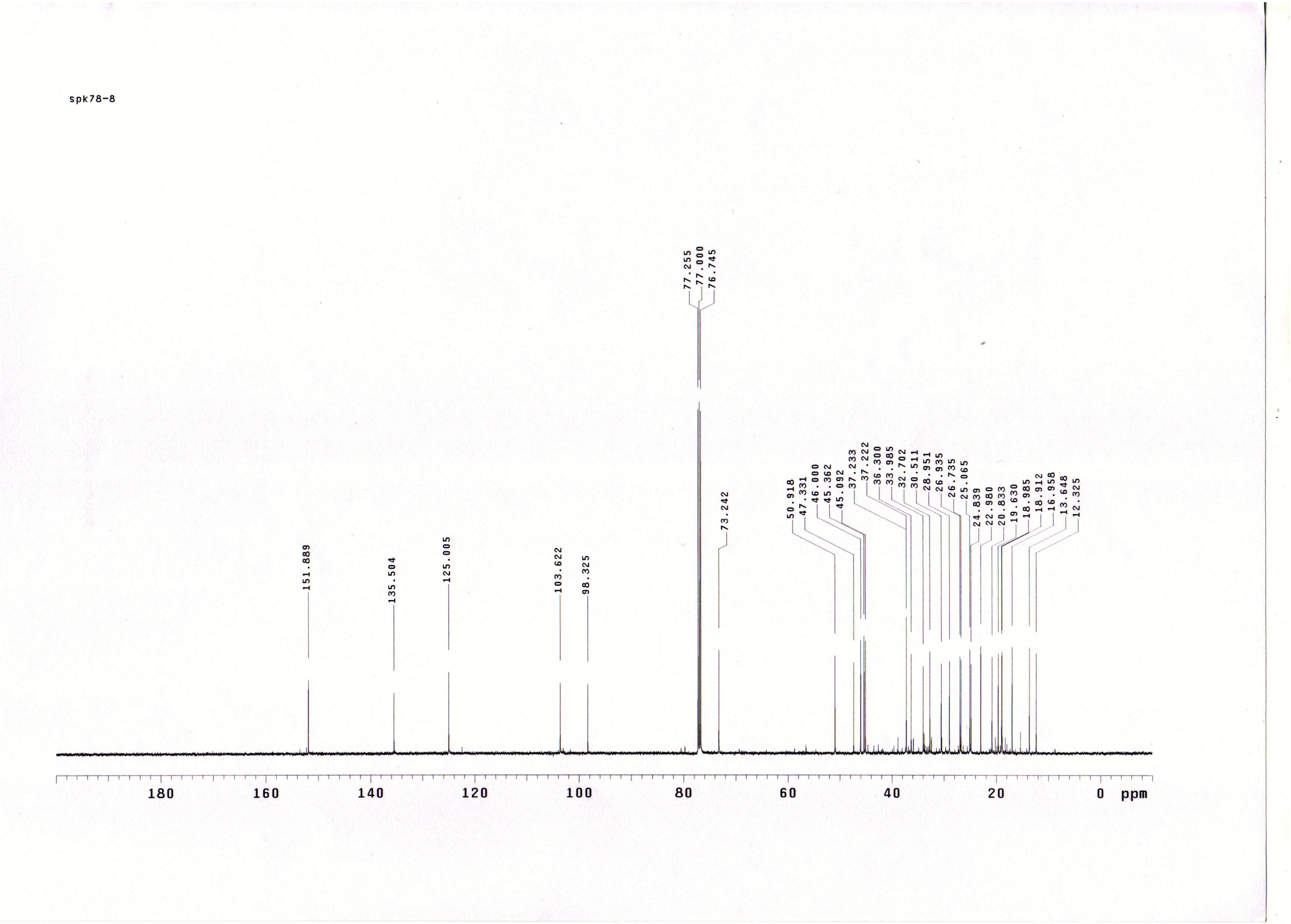

S3. ^{13}C NMR spectrum of **1** in $CDCl_3$ at 125 MHz.

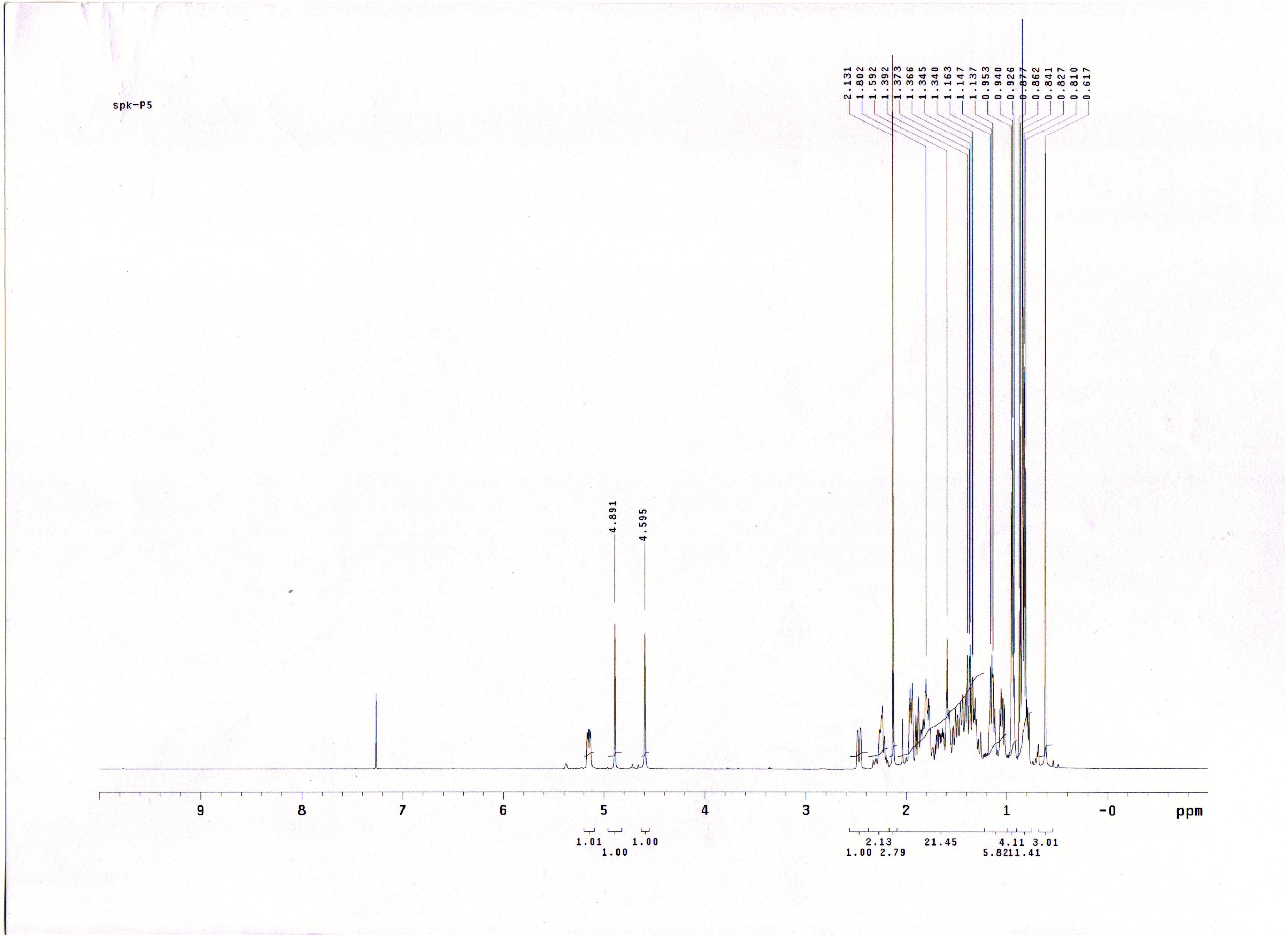

S4. ^{1}H NMR spectrum of **2** in $CDCl_3$ at 500 MHz.

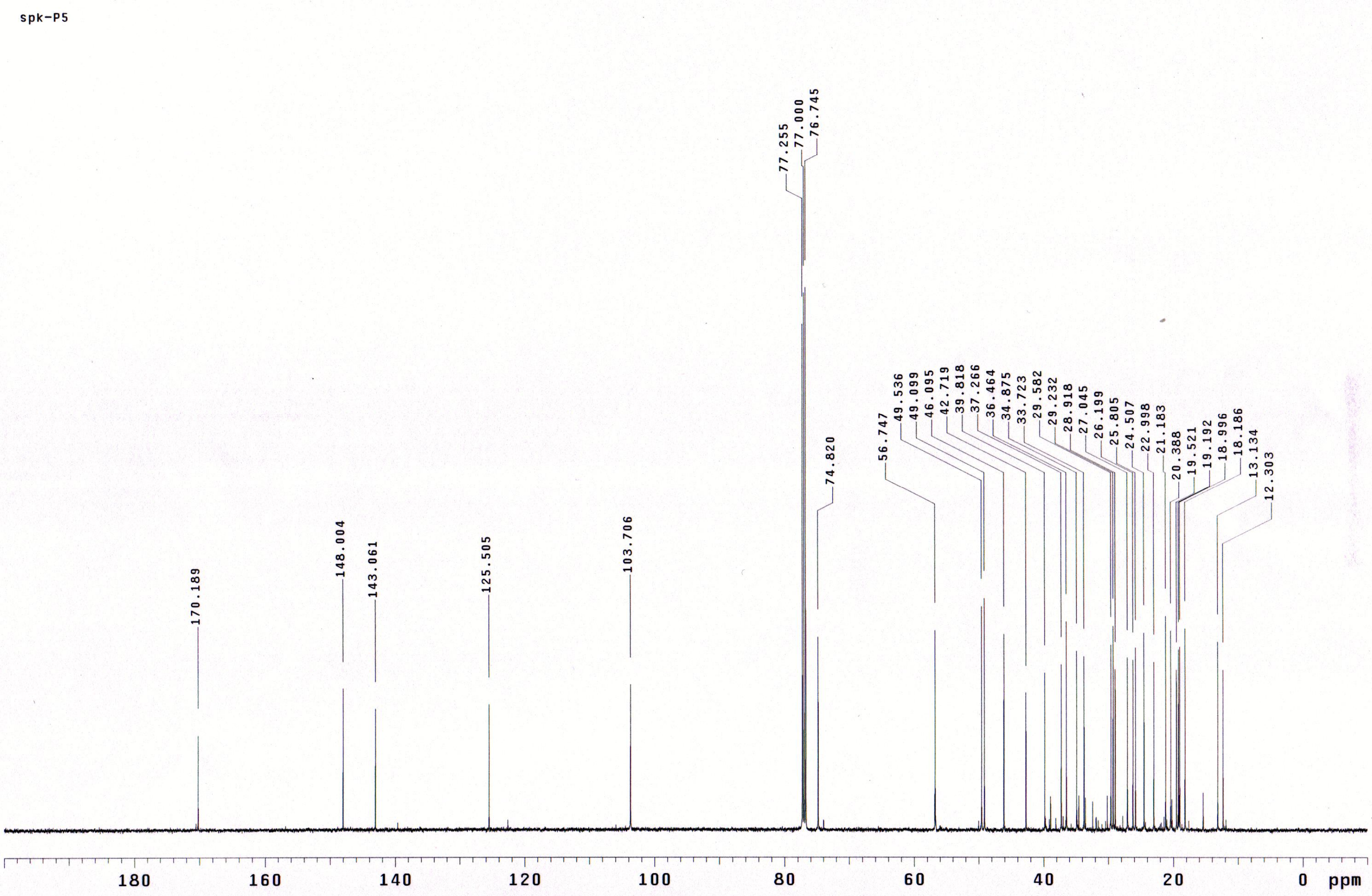

S5. ^{13}C NMR spectrum of **2** in $CDCl_3$ at 125 MHz.

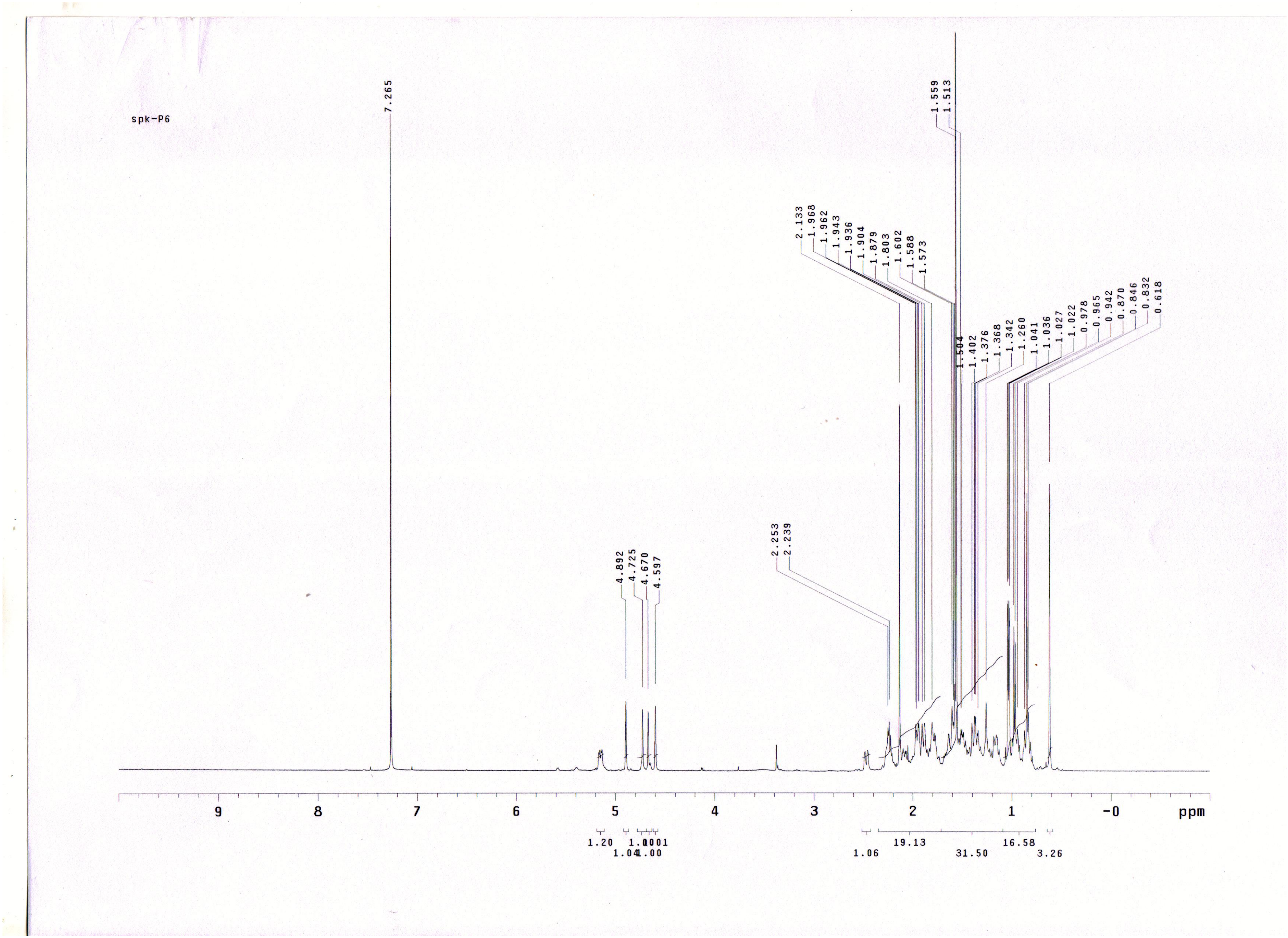

S6. ^{1}H NMR spectrum of **3** in $CDCl_3$ at 500 MHz.

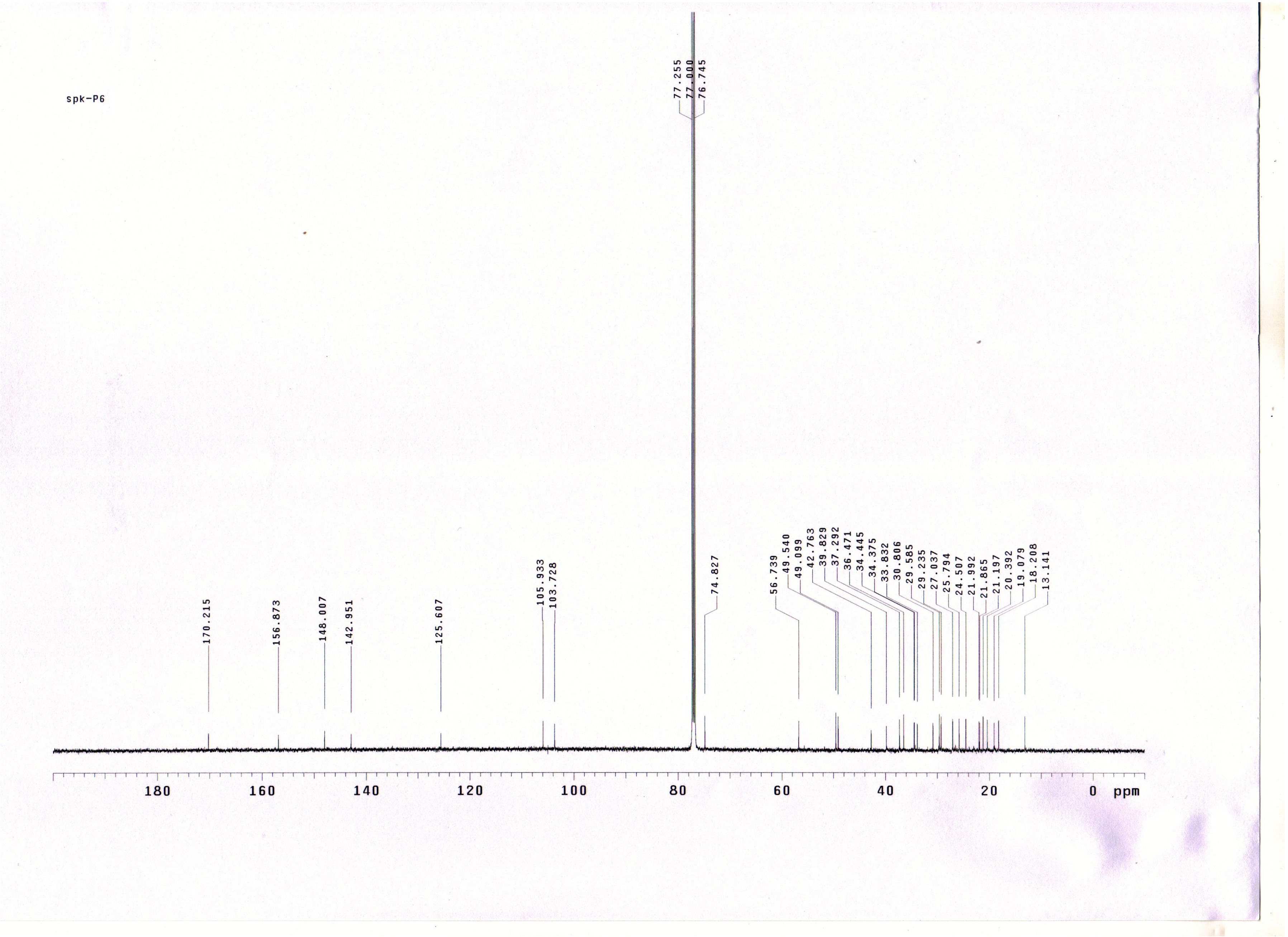

S7. ^{13}C NMR spectrum of **3** in $CDCl_3$ at 125 MHz.

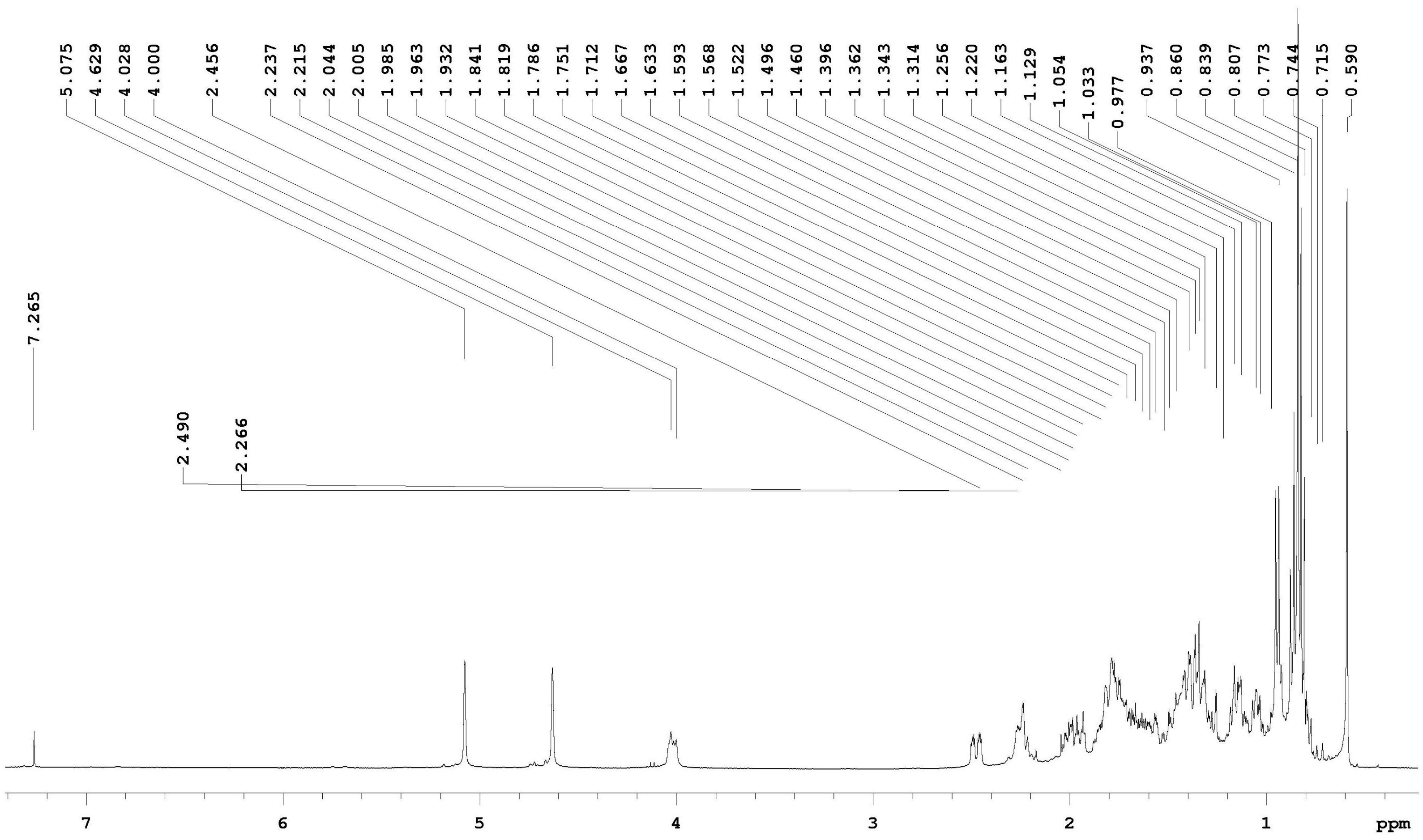

S8. ^{1}H NMR spectrum of **4** in $CDCl_3$ at 400 MHz.

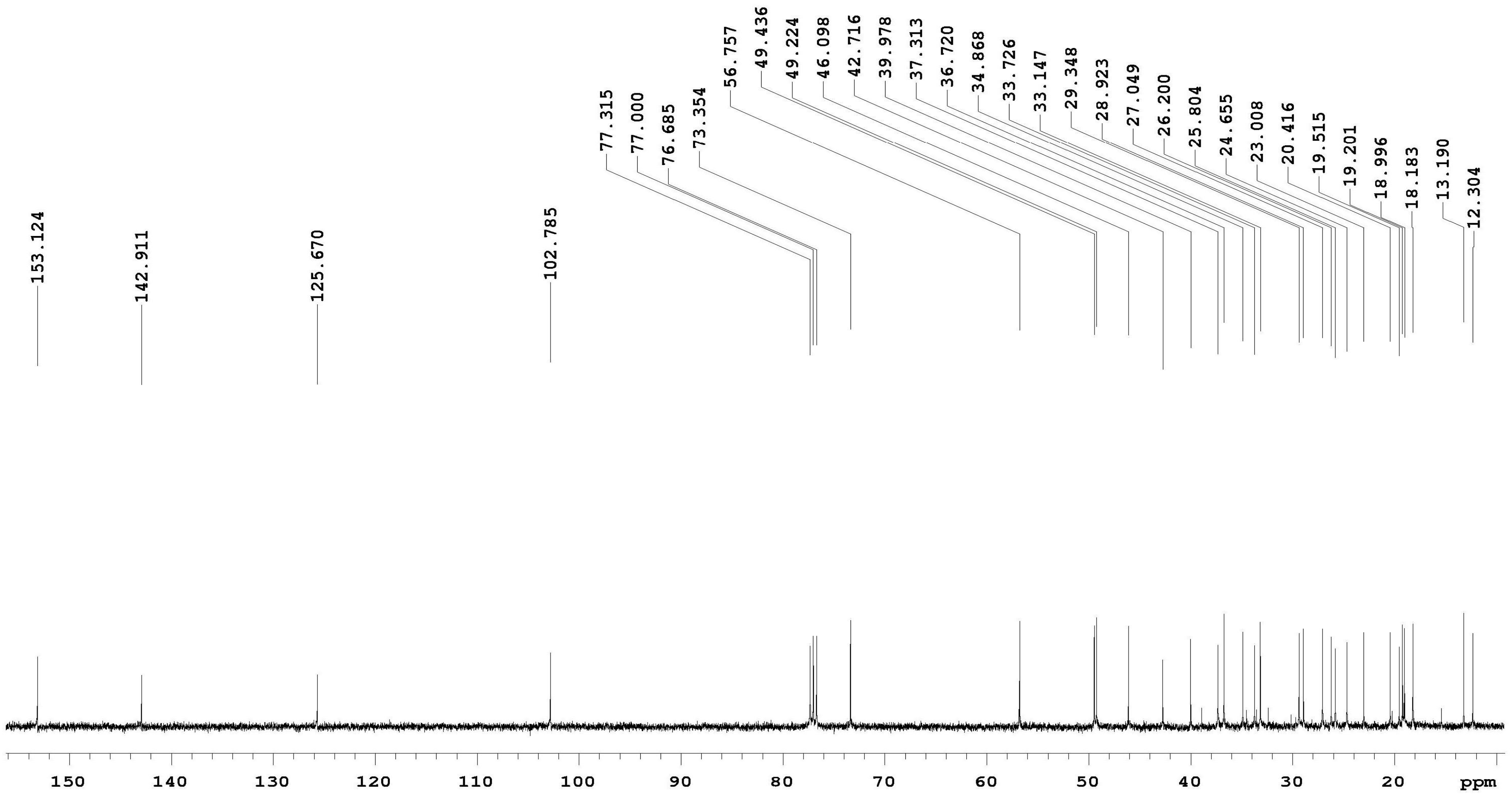

S9. ^{13}C NMR spectrum of **4** in $CDCl_3$ at 100 MHz.

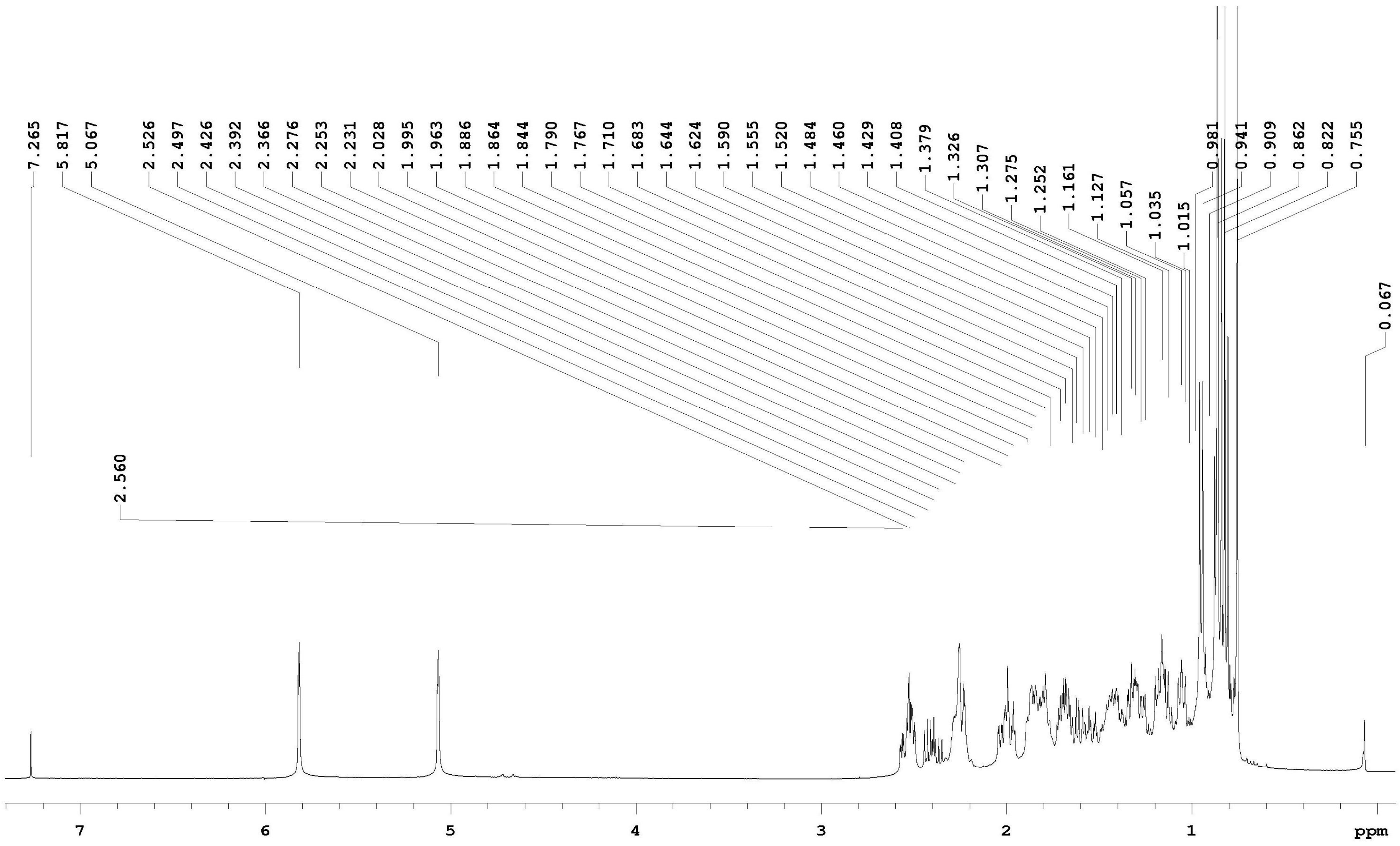

S10. ^{1}H NMR spectrum of **5** in $CDCl_3$ at 400 MHz.

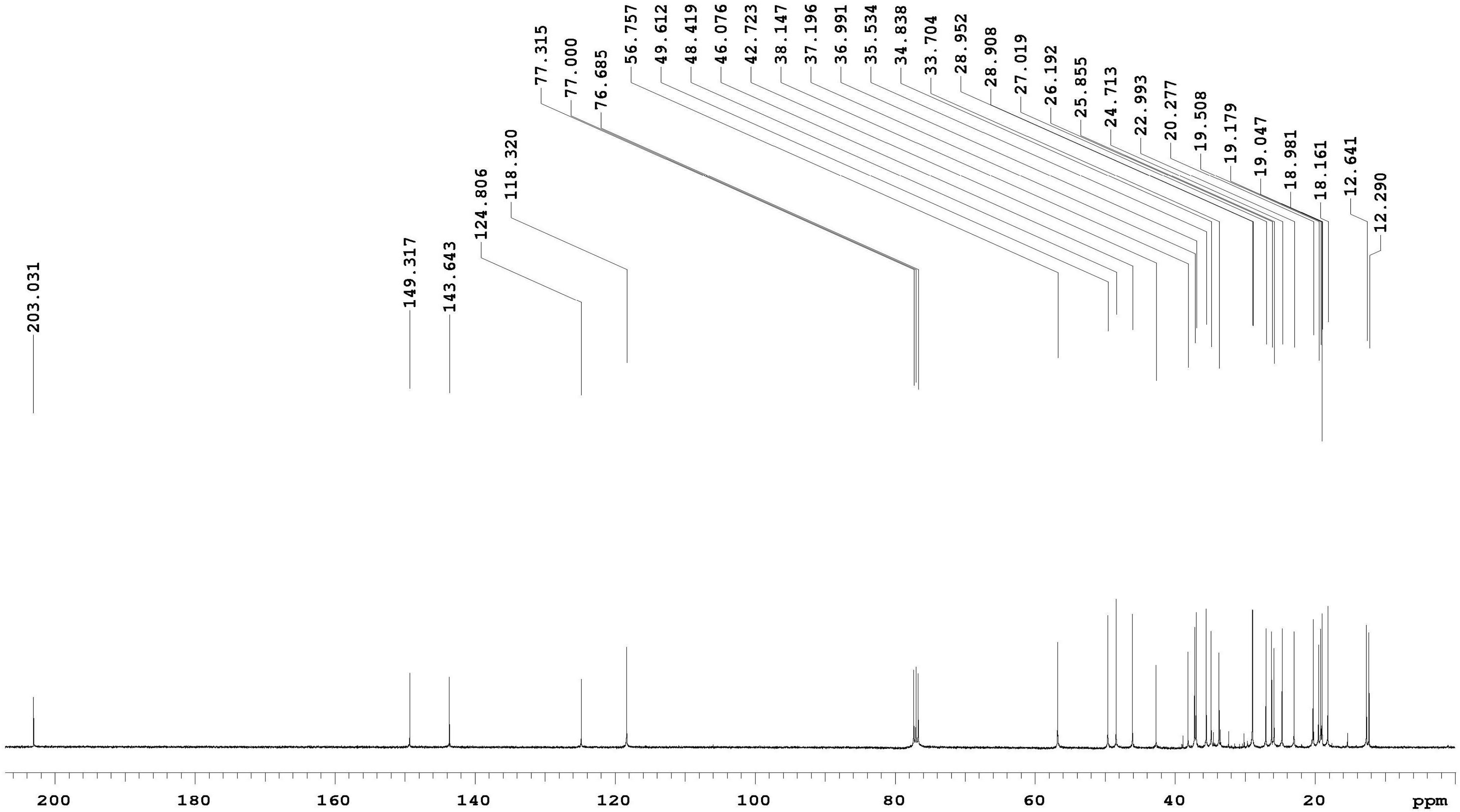

S11. ^{13}C NMR spectrum of **5** in $CDCl_3$ at 100 MHz.
